# Needle Arthroscopic Subchondroplasty With Adipose-Derived Stem Cell Augmentation for the Treatment of Osteochondral Lesions of the Talus

**DOI:** 10.1016/j.eats.2023.05.014

**Published:** 2023-09-04

**Authors:** Jari Dahmen, Cristian Indino, Riccardo D’Ambrosi, Federico G. Usuelli

**Affiliations:** a University of Amsterdam, Department of Orthopedic Surgery and Sports Medicine, Amsterdam, The Netherlands; b Amsterdam Movement Sciences, Musculoskeletal Health, Sport, Amsterdam, The Netherlands; c Academic Center for Evidence-based Sports Medicine, Amsterdam, The Netherlands; d Amsterdam Collaboration for Health & Safety in Sports, International Olympic Committee Research Center, Amsterdam, The Netherlands; e Ortopedia della Caviglia e del Piede, Humanitas S. Pio X, Milano, Italia; f Istituto di Ricovero e Cura a Carattere Scientifico, Ospedale Galeazzi–Sant’Ambrogio, Milano, Italia; g Università degli Studi di Milano, Dipartimento di Scienze Biomediche per la Salute, Milano, Italia

## Abstract

Needle arthroscopy has enjoyed a tremendous growth concerning the quality of intraoperative images due to technical innovation, resulting in innovative possibilities concerning concomitant minimally invasive procedures and treatment of osteochondral lesions of the talus (OLT). These lesions have increasingly been receiving scientific attention in the orthopaedic (sports) medicine field, and, as such, the quality of evidence-based treatment for them has developed substantially. Treatment of OLTs—and specifically subchondroplasty. OLTs may also be suitable for needle arthroscopic interventions. The purpose of the present technical note is, therefore, to present an all-arthroscopic needle arthroscopic technique, including subchondroplasty with adipose-derived stem cells augmentation for osteochondral lesions of the talus.

Osteochondral lesions of the talus (OLTs) are characterized by damage to the talar articular cartilage and the underlying subchondral bone. There is no superior treatment for primary OLTs.^1^ The specific choice for the intervention in question depends on lesion factors, such as the primary or nonprimary nature of the lesion, lesion dimensions, and lesion morphology. One treatment option that has gained popularity over the past decade is the usage of Subchondroplasty, which includes the injection of a flowable nanocrystalline calcium phosphate synthetic bone graft into the subchondral region of the talar bone. An endothermic reaction takes place thereafter, thereby, crystallizing the calcium phosphate to mimic the properties of cancellous bone. The receiving bone site will then resorb the osteoconductive calcium phosphate over in order to replace it with native cancellous bone.^2^ A potential addition to the treatment may be a biological augmentation through the usage of adipose-derived stem cells.^3^^,^^4^ Moreover, in order to minimize the invasiveness of the procedure, the application of minimally invasive smaller arthroscopes may be of benefit. As such, a 2-mm bendable needle arthroscope has been developed and proven to be safe and applicable for different indications.^5^^,^^6^ The management of OLTs can, therefore, also be suitable for needle arthroscopic interventions. It is, therefore, the purpose of the technical note to present an all-arthroscopic needle arthroscopic technique, including subchondroplasty with adipose-derived stem cells augmentation for osteochondral lesions of the talus.

## Ethical Approval

The study was conducted in agreement with the 1964 Helsinki Declaration and its later amendments. Ethical approval by the institution’s review board was not required.

### Surgical Technique

The procedure is demonstrated in Video 1 using a step-by-step approach. The technique is demonstrated in the current article using intraoperative material.

### Preoperative Planning

The preoperative planning consists of a proper physical examination taking into account the patient’s wishes and will be followed by a shared-decision making process by the treating surgeon. The preoperative magnetic resonance imaging (MRI) reports are of importance to confirm the diagnosis and accurately study the anatomy of the subchondral cyst in order to determine the amount of subchondroplasty (SCP, Zimmer Holdings, Inc., Warsaw, IN) to be injected.

### Equipment and Patient Setup

As equipment, we use the needle arthroscopic device (NanoScope, Arthrex, Naples, FL), which consists of a sterile disposable handpiece set and the portable video console.^7^ The patient is positioned supinely with the abdomen and flanks freely exposed with the patient under full anesthesia. The locations of the portal placement and the surface anatomy are marked out.^7^ A tourniquet is applied at the thigh and is inflated to 250 mmHg. The surgical field is disinfected with a chlorohexidine solution and covered with sterile draping.

### Adipose-Derived Stem Cell Augmentation: Emulsification, Harvesting, and Processing

**Fig 1:**
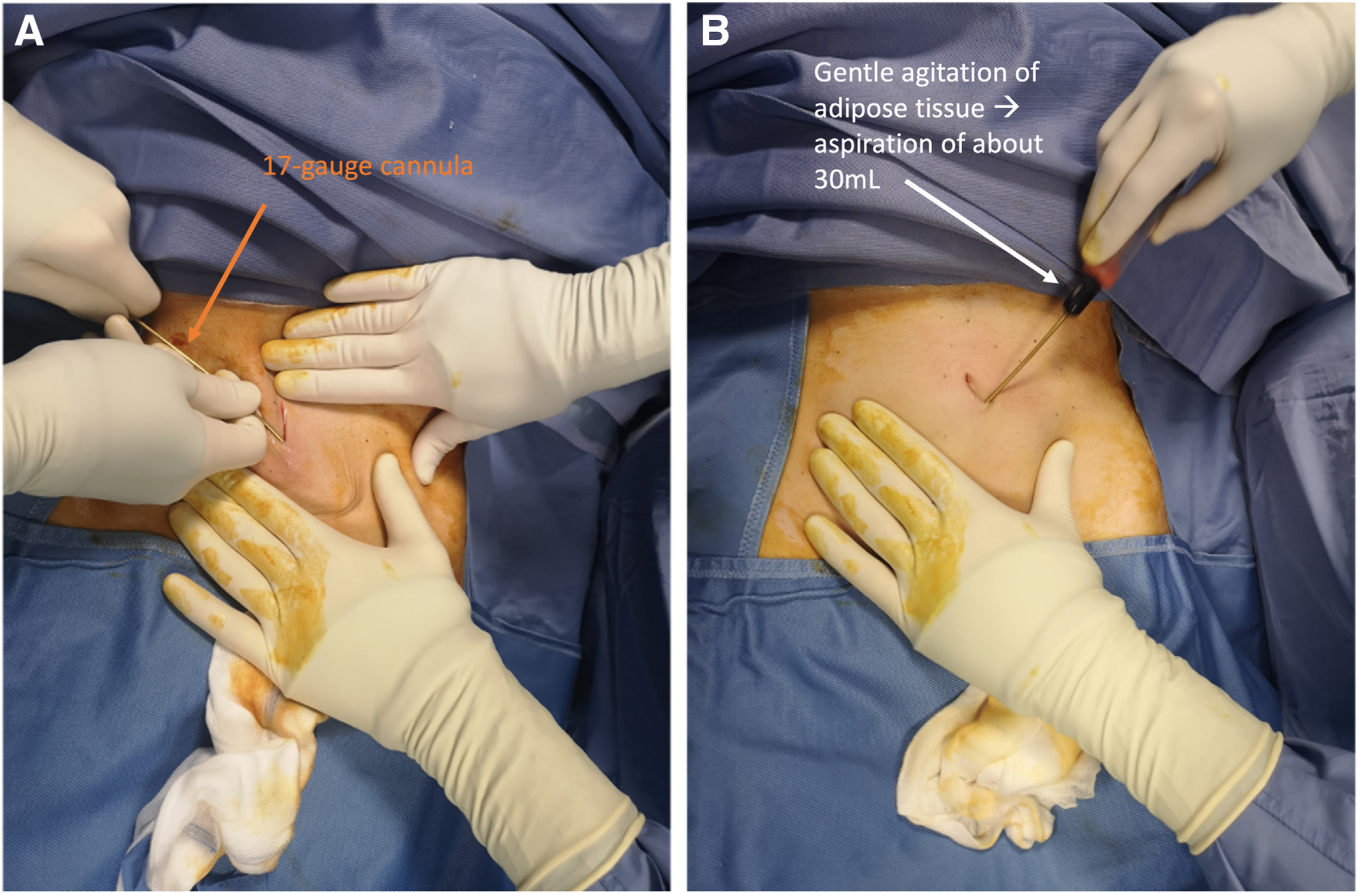
(A) A tumescent solution composed of 500 mL of saline with 40 mL of 2% lidocaine and 0.5 mL of adrenaline/epinephrine was injected subcutaneously using a 17-gauge cannula both below and above the umbilicus. (B) Gentle agitation of the adipose tissue through a hacking tapotement technique was performed, which was followed by an aspiration of adipose tissue of 30 mL.

**Fig 2:**
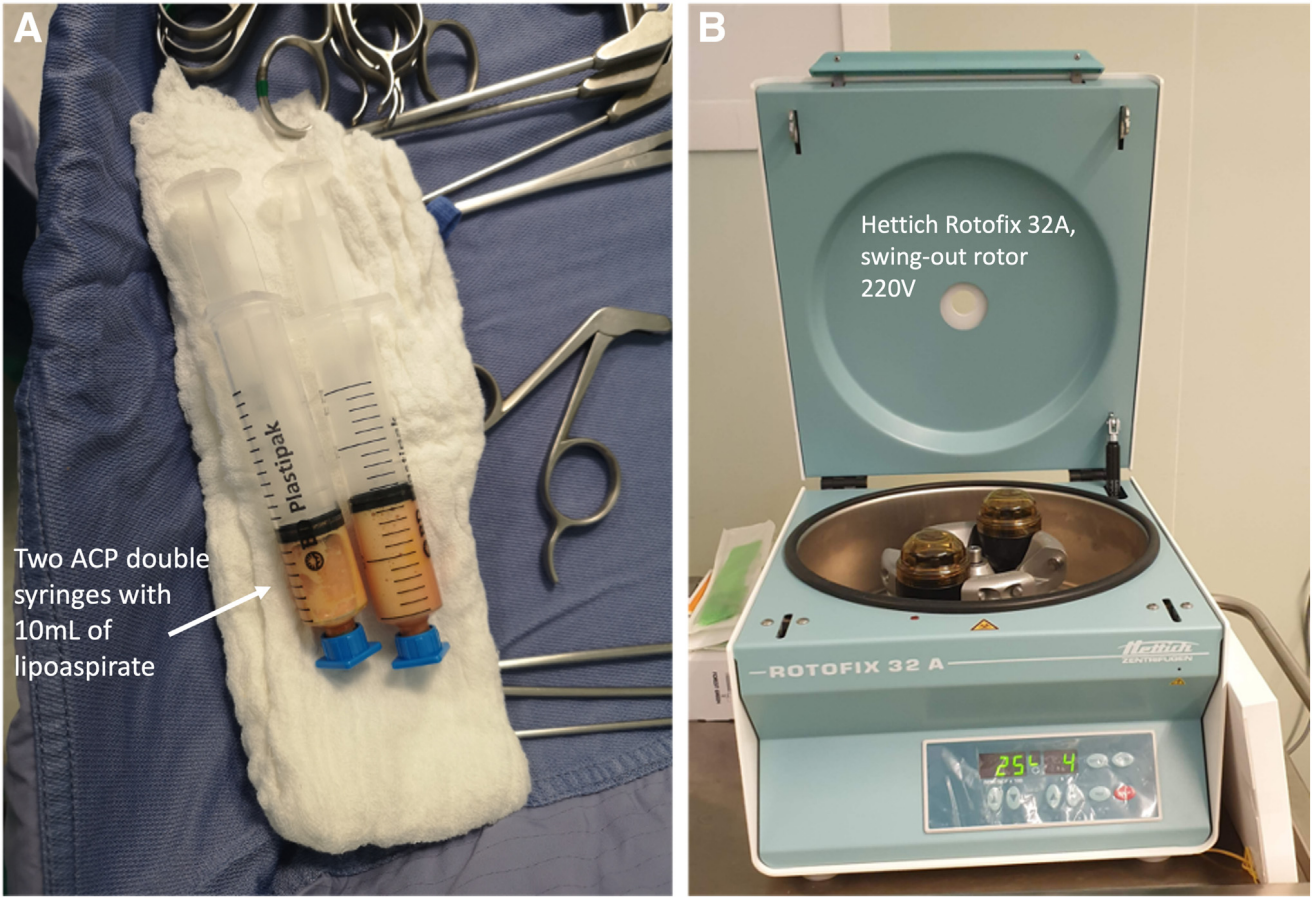
(A) Two ACP double syringes with approximately 10 mL of lipoaspirate prior to centrifuging at 2,500 rpm, 5 minutes at room temperature. (B) Hettich Rotofix 32A, swing-out rotor, 220V centrifuge system.

The procedure is initiated by the adipose-derived stem cell augmentation process consisting of emulsification, harvesting, and processing (Video 1). The abdominal harvesting site is prepared and draped in a sterile fashion with disposable drapes and chlorhexidine as per sterility protocol. An umbilical incision is performed through a stab incision in the subcutaneous tissue. Thereafter, a tumescent solution composed of 500 mL of saline with 40 mL of 2% lidocaine and 0.5 mL of adrenaline/epinephrine is injected subcutaneously using a 17-gauge cannula both below and above the umbilicus (Fig 1). Gentle agitation of the adipose tissue through a hacking tapotement technique is performed followed by an aspiration of adipose tissue of 30 mL. 10-15 mL of lipoaspirate is transferred to two ACP syringes using a 2.4-mm transparent transfer adapter for micro fat (Arthrex, Munich, Germany). The ACP double syringes filled with 10-15 mL of decanted lipoaspirate are transferred to the centrifuge system (Hettich Rotofix 32A, Swing-out rotor, 220V) and are subsequently centrifuged at 2,500 rpm for a total number of 4 minutes at room temperature (Fig 2). The condensed lipoaspirate is microfragmented through swooshing it 40 times forward and backward over the transfer system, after which a second round of centrifugation takes place eventually yielded 2-3 mL of condensed concentrated adipose tissue. The abdominal incision is closed thereafter.

### Subchondroplasty Under Nanoarthroscopic and Radiographic Control

**Fig 3:**
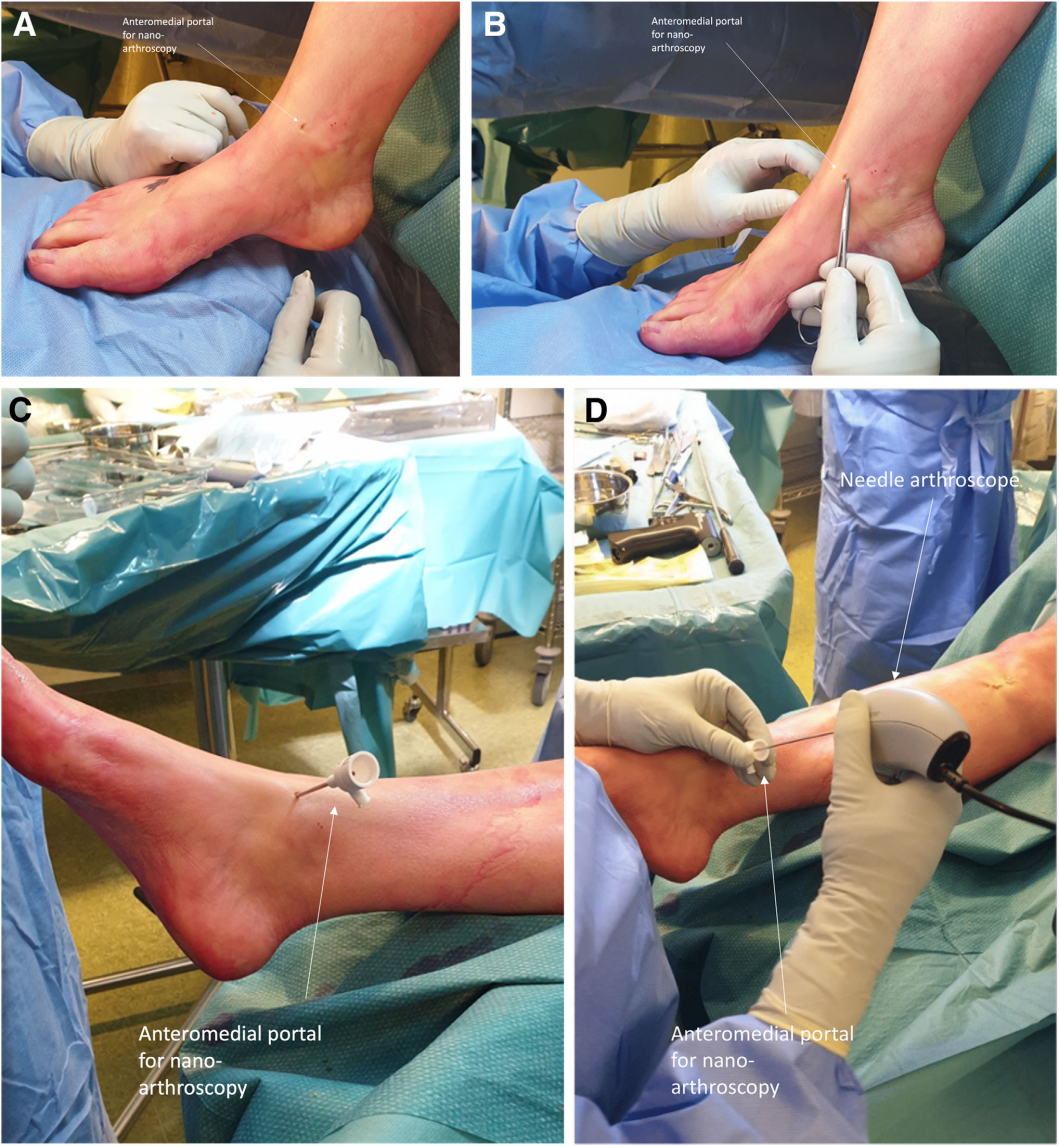
(A and B) Needle arthroscopic approach to the left ankle via an anteromedial approach; the anteromedial portal can be used as primary vision portal and the anterolateral portal can be used as primary intervention portal. (C and D) However, the portals can be switched interchangeably in order to create adequate vision and create an easy approach to reach the medial or lateral part of the ankle joint and perform the treatment accordingly.

**Fig 4:**
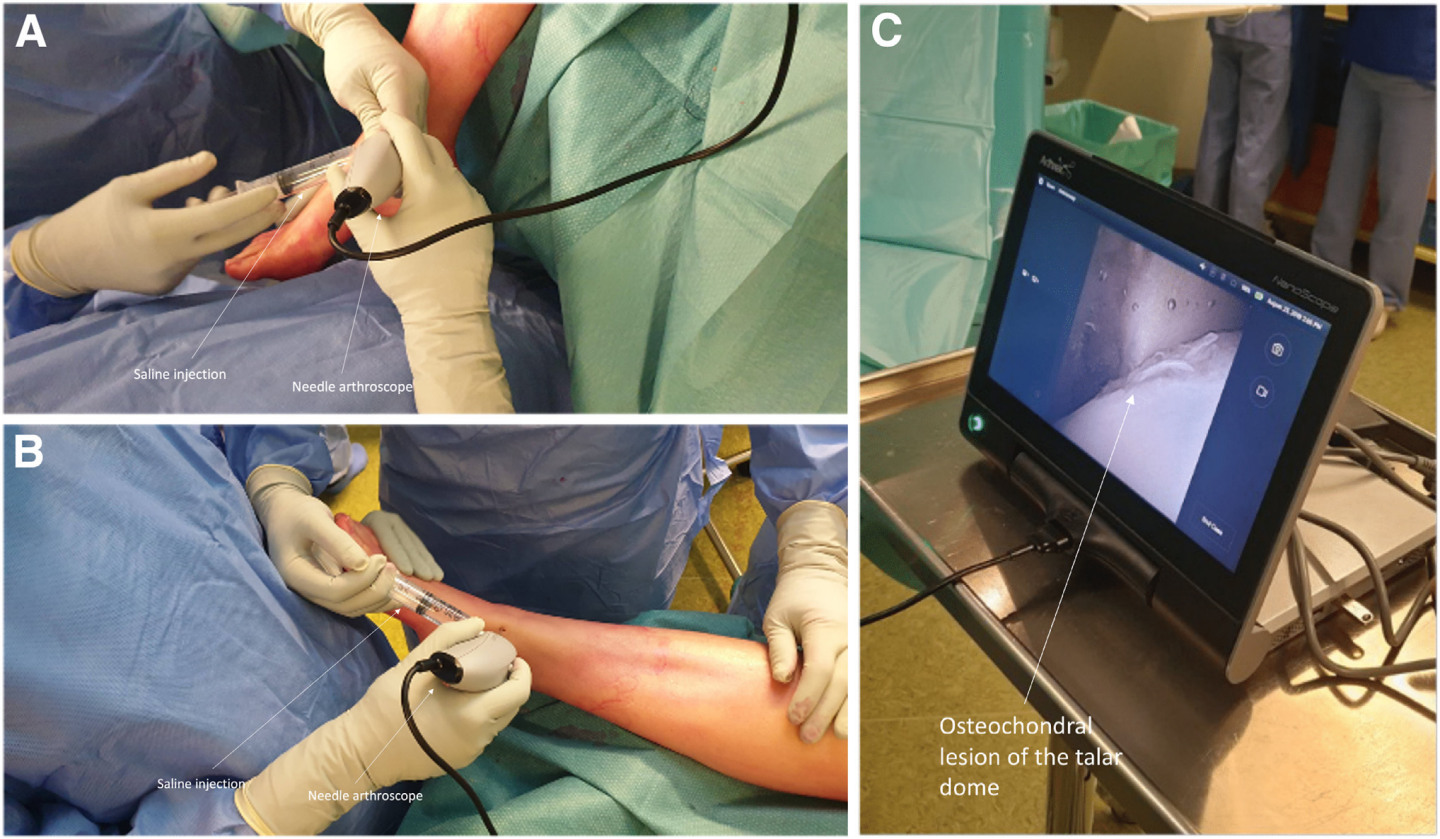
(A and B) The needle arthroscopic approach is continued by connecting the syringe directly to the sheath of the needle arthroscopy in order to distend the joint with sterile saline; an assistant can assist with this by manually and directly injecting the saline appropriately. (C) Afterward, the joint is then assessed thoroughly during which the osteochondral lesion can be observed.

**Fig 5:**
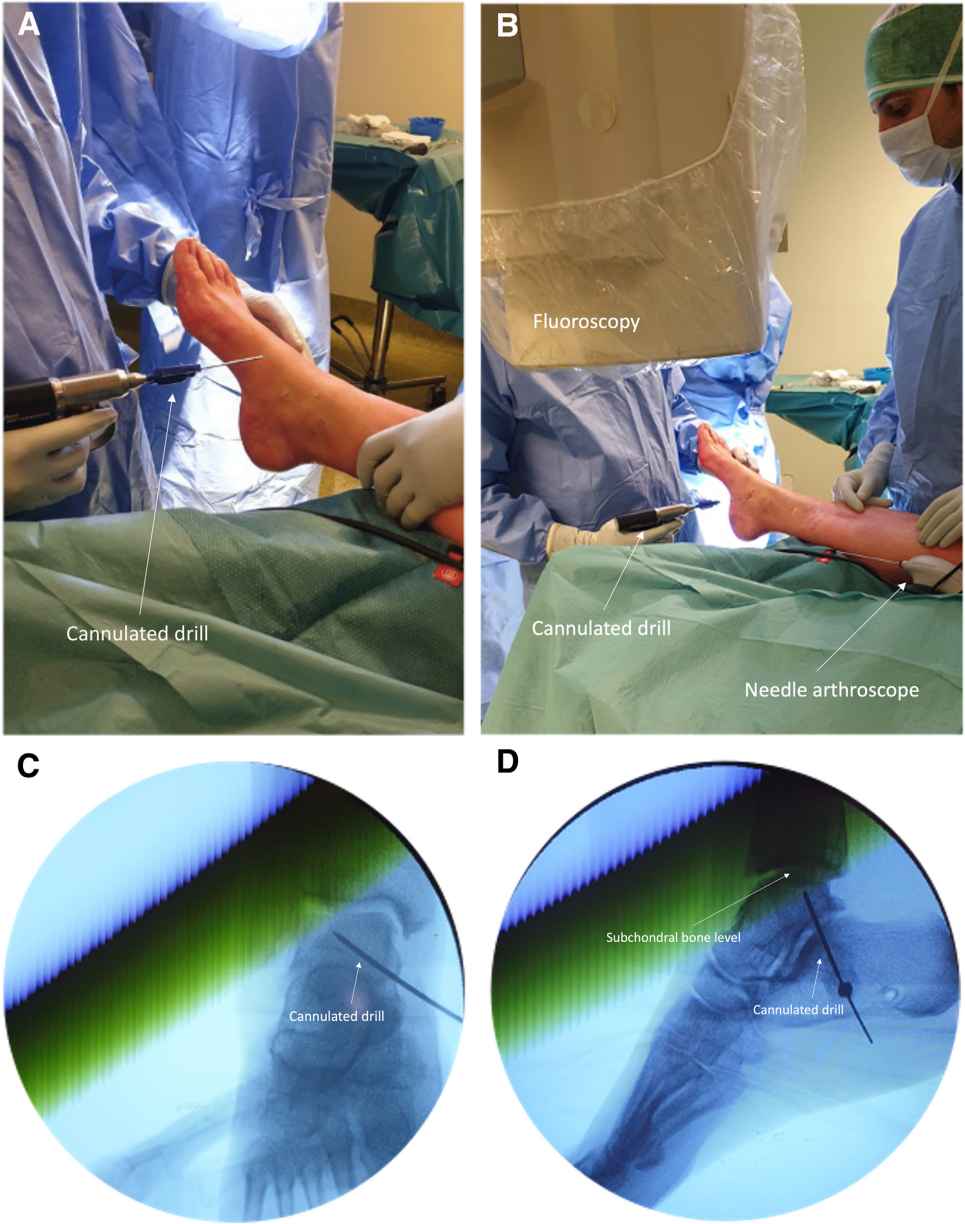
(A and B) A cannulated drill is used to drill the subchondral cyst, which is performed under fluoroscopic guidance and control. (C and D) The fluoroscopy is used in order to check whether the drilling is at an adequate point, namely, just below the subchondral bone plate. This is done in order to verify the position of the potential insertion of the subchondroplasty.

**Fig 6:**
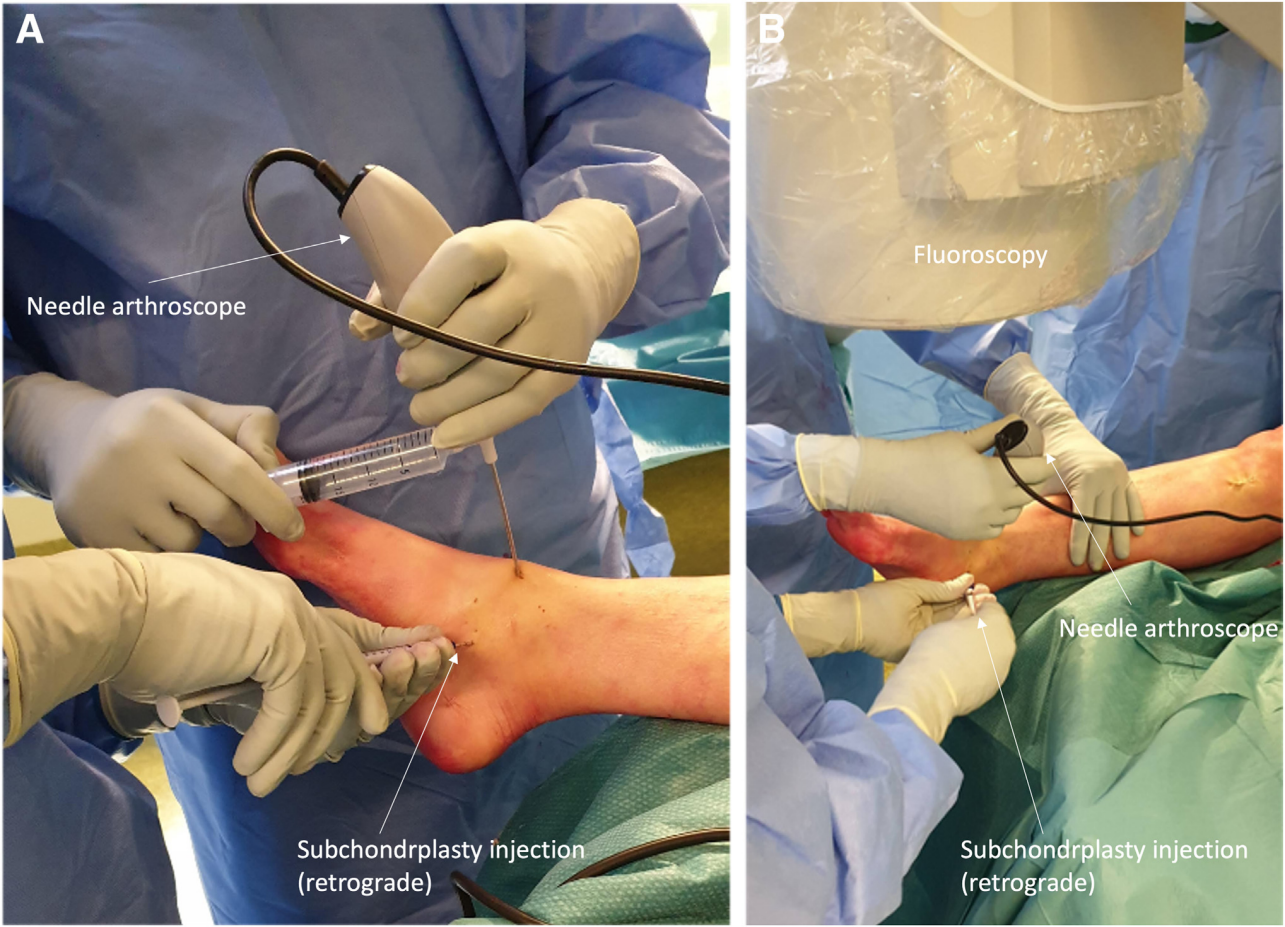
After having drilled the cannulated rill through the subchondral cyst under fluoroscopic guidance and control, and 5 mL of subchondroplasty material are injected into the subchondral lesion area (also under both fluoroscopic guidance and control) using the needle-arthroscopic procedure to verify that there is no leakage of the subchondroplasty into the joint after full insertion. (A and B) After this, the concentrated adipose-derived stem cells are injected in the ankle under nano-arthroscopic control.

An arthroscopic system (NanoScope, Arthrex, Naples, FL) is used consisting of a disposable handpiece and a control unit consisting of a screen. The details of the arthroscopic system are described in earlier publications by Stornebrink et al.^5^ In order to obtain portal access, 2-mm skin incisions are made parallel to the relevant surrounding anatomic structures after which the anteromedial and anterolateral portals are created, as described by Golano et al.^8^ (Table 1) (Fig 3). The whole joint is inspected with the Nanoscope through the stepwise description of Vega et al.^9^ The cartilage layer is assessed in all compartments to determine any kind of lesions and is graded according to the recommendations of the International Cartilage Repair Society on the 9-grid scheme^10^ (Fig 4). Then, attention is shifted toward the subchondroplasty procedure; on the basis of the preoperative MRI images and the calculated volume of the cyst(s), a cannulated drill is used to drill the subchondral cyst, which is performed under fluoroscopic guidance and control (Fig 5). Five milliliters of subchondroplasty material are injected into the subchondral lesion area under both fluoroscopic guidance and control, as well using the needle-arthroscopic procedure to verify that there is no leakage of the subchondroplasty into the joint after full insertion (Fig 6). Thereafter, the concentrated adipose-derived stem cells are injected in the ankle under nanoarthroscopic control (Fig 6).

**Table 1:** Indications and Contraindications

Indications	Contraindications
Primary and nonprimary symptomatic osteochondral lesions of the talus	Presence of big osteophytes to the ankle joint preventing an adequate introduction of the instruments, as well as a proper visualization of the joint
Symptoms for at least 6 months and having followed and adequate conservative treatment protocol	Decreased or inadequate vascular status
Deep ankle pain during walking/weight-bearing, which decreases during rest	No adequate conservative protocol followed yet
	Infections; peripheral arterial occlusive disease
	Critical soft-tissue conditions / compromise
	Severe joint-space narrowing

### Closure

All instruments are removed, and sterile wound closure strips or a simple band aid are sufficient for skin closure of the percutaneous camera portal. A wire suture is used for the working portal. Standard postoperative rehabilitation is started.

## Discussion

The needle arthroscopic technique combined with a subchondroplasty technique, biologically augmented with adipose-derived stem cells, as presented in the present article provides a minimally invasive surgical approach to treat osteochondral lesions of the talus of cystic nature. The advantages of this procedure are the minimal invasiveness of the needle arthroscopy, the potentially improved postoperative recovery due to the decreased soft-tissue trauma and potentially improved patient experience, while the advantages of the biological augmentation through the adipose-derived stem cell therapy may stimulate a greater subchondral bone and cartilage recovery.^3^^,^^4^ Recently, needle arthroscopy has been reintroduced in the orthopaedic field, as the quality of the images substantially improved over the past 5 years.^5^^,^^7^^,^^11^^,^^12^ As a result, the technique is increasingly being applied for both diagnostic and concomitant interventional procedures of minimally invasive nature.^5^ We expect that the body of evidence on the cost-effectiveness, as well as clinical efficacy and sensitivity and specificity, in relation to radiological reports, will grow over the coming years. Concerning the usage of subchondroplasty, one can state that currently, there is limited evidence on the clinical efficacy of the usage of subchondroplasty for OLTs, also, there are some reports that have shown an avascular necrosis of the talar bone after the usage and application of subchondroplasty.^2^ Therefore, one should be careful with the described technique and solely apply it in selected cases, taking into account the potential pearls, pitfalls, and disadvantages (Table 2) through a high-quality shared-decision making process and after a thorough discussion of the procedure with the patient.

**Table 2:** Pearls and Pitfalls

Tips and Pearls	Pitfalls
Tumescent solution injection followed by hacking tapotement technique to promote emulsification	Swelling and ecchymosis minimized by application of abdominal binder for 2 days postoperatively
Permit sedimentation of stem cells to decant excess fluid	Preoperative screen for abdominal hernias and scars to avoid injury to abdominal contents during lipoaspiration
Drain arthroscopy fluid before stem cell injection	Sequential harvest site order inpatients with low percentage body fat: below umbilicus, above umbilicus, flanks, and buttocks.
	Incorrect portal placement, causing iatrogenic (neurovascular) damage to the peroneal superficial nerve.
Careful placement of the lateral portal under direct arthroscopic visualization.	To avoid postoperative stiffness of the ankle joint, prolonged postoperative immobilization should be avoided.
Decreased postoperative pain levels due to minimal invasiveness of the procedure.	Iatrogenic damage to the articular cartilage of the ankle joint from (incorrect) trocar placement.
The needle arthroscopic procedure provides a minimally invasive alternative to conventional arthroscopy for the treatment of (osteo)chondral injuries of the ankle joint as such enhancing the postoperative rehabilitation and joint stiffness.	

As a conclusive remark, needle arthroscopy, combined with a subchondroplasty and a subsequent biological augmentation with adipose-derived stem cells, provides a minimally invasive, high-quality cartilage repair technique that can increase patient-reported outcome measures and improve the postoperative recovery due to decreased soft-tissue trauma and potentially improved patient experience.

## Supplementary Data

ICMJE author disclosure forms

Video 1 The authors describe the technique for needle arthroscopic inspection and treatment of (osteo)chondral lesions of the ankle joint as a standardized approach. The osteochondral lesions of the ankle joint can be located at any talar location and are preferably nonfragmentous and of size <1.5 cm. It must be stated that the lesion should be of symptomatic nature and have already undergone a rigid and thorough conservative treatment approach prior to the surgery. In the video, the equipment is noted, including sterilization, the set of the needle arthroscopy, including a handpiece set, a portable console, 50 cc syringes, and a 3-way tap, as well as sterile sodium chloride 0.9%. Besides the needle arthroscopic set, a clear description of the harvesting of stem cells from the adipose tissue is given. After this harvesting, the aspirated tissue is centrifuged with a Hettich Rotofix 32A swing-out 220V centrifuge system. The procedure that is described is then continued by the application of subchondroplasty: a cannulated drill is used to drill the subchondral cyst, which is performed under fluoroscopic guidance and control. Five milliliters of subchondroplasty material are injected into the subchondral lesion area under both fluoroscopic guidance and control, as well using the needle-arthroscopic procedure to verify that there is no leakage of the subchondroplasty into the joint after full insertion. Thereafter, the video describes the injection of the concentrated adipose-derived stem cells in the ankle under nanoarthroscopic control.
